# Biomimetic Metal-Organic Framework Nanoparticles for Synergistic Combining of SDT-Chemotherapy Induce Pyroptosis in Gastric Cancer

**DOI:** 10.3389/fbioe.2022.796820

**Published:** 2022-02-21

**Authors:** Zhu Yu, Wenlong Cao, Chuangye Han, Zhen Wang, Yue Qiu, Jiancheng Wang, Mengda Wei, Junfu Wang, Siwen Zhang, Senfeng Liu, Shutian Mo, Junqiang Chen

**Affiliations:** ^1^ Department of Gastrointestinal Surgery, The First Affiliated Hospital of Guangxi Medical University, Nanning, China; ^2^ Guangxi Key Laboratory of Enhanced Recovery After Surgery for Gastrointestinal Cancer, Nanning, China; ^3^ Department of Hepatobiliary Surgery, The First Affiliated Hospital of Guangxi Medical University, Nanning, China

**Keywords:** ZIF-8, sonodynamic therapy, chemotherapy, gastric cancer, pyroptosis

## Abstract

In recent years, sonodynamic therapy (SDT) has been widely developed for cancer research as a promising non-invasive therapeutic strategy. Here, we synthesized zeolitic imidazole frameworks-8 (ZIF-8) and utilized its properties to encapsulate hydrophobic Chlorin e6 (Ce6) and hydrophilic tirapazamine (TPZ) for a synergistic sonodynamic chemotherapy, which was also accompanied by the modification of cytomembrane of gastric cancer (GC) cells. Thus, we enabled the biomimetic property to achieve targeted delivery. Ce6-mediated SDT, in combination with ultrasound irradiation, could target the release of reactive oxygen species (ROS) to aggravate further hypoxia and activate TPZ. Combining these effects could induce the pyroptosis of GC cells and play the anti-tumor function, which could provide a potential therapeutic method for cancer therapy.

## Introduction

Every year, over one million new cases of gastric cancer (GC) are diagnosed. Also, GC has already become the fifth most common cancer globally (Sung et al., 2021). The mortality in gastric cancer is high, and usually, it is diagnosed at an advanced stage, which makes it rank the fourth for mortality (Smyth et al., 2020; Sung et al., 2021). The main therapies for gastric cancer cover surgery, chemotherapy, radiotherapy, chemoradiotherapy, immunotherapy, and molecular-targeted therapy (Song et al., 2017; Johnston and Beckman, 2019). Despite these therapies, the 5-year overall survival rate remains low, with an approximate rate of 20% in most countries of the world (Correa, 2013). Hence, a new potential therapeutic strategy for gastric cancer is yet to be explored.

Ce6 is a kind of organic sonosensitizer with minimal side effects, which can be better accumulated in tumors and also can be eliminated faster from the organisms. It is a type of porphyrin that has properties of high sensitization with the capability to produce ROS (Rengeng et al., 2017). Ce6 is reported to be activated by light/ultrasound and can inhibit the growth of tumor cells (Li et al., 2014; Bhatta et al., 2019). However, Ce6 has a property of hydrophobicity in the water environment, and since it gathers into a bigger crystal, it gets harder for it to function completely (Liu et al., 2017; Zhang et al., 2018). Also, it can be cleared and degraded more rapidly in long-term blood circulation (Liu et al., 2017). Hence, the question arises: how do we find a good way to deliver Ce6 for it to play an anti-tumor function?

Hypoxia or severe oxygen deprivation is considered as one evident challenge in the therapy of solid tumors because hypoxic regions are against most anti-tumor drugs preventing the killing of tumor cells (Marcu and Olver, 2006; Guo et al., 2020). Tirapazamine (TPZ), a new class of bioreductive cytotoxic drugs, can be selectively toxic to hypoxia cells (Gandara et al., 2002; Reddy and Williamson, 2009). Due to the lack of oxygen within the cells, TPZ can be reduced to toxic-free radicals that can induce breakages of cellular DNA (Gatzemeier et al., 1998). TPZ has been put into use in phase II and III clinical studies, and studies have proven that the combined use of TPZ and cisplatin could enhance the anti-tumor effect in malignant and non-small cell lung cancers (Denny and Wilson, 2000; Marcu and Olver, 2006). Therefore, we should take the advantage of the hypoxic environment of a tumor to maximize the anti-tumor effects.

ZIF-8 is a member of the metal-organic frameworks (MOFs) synthesized using Zn^2+^ ion and 1,2-methylimidazolate (1,2-MIL) (Lu et al., 2012). It is known for its high surface area, tunable porosity, and excellent chemical and thermal stability. It is also easy to synthesize it in water and alcoholic phases (Wu et al., 2015). In particular, it has a pH-responsive structure, which indicates its ease of degradation in an acidic environment along with the maintenance of steadiness in neutral conditions (Cai et al., 2019; Cheng et al., 2019). Due to these properties, ZIF-8 can be used as a framework to load drugs and target the release of drugs according to its response against the pH of the acidic tumor microenvironment (An et al., 2020b). Importantly, due to the strong electrostatic interaction of Ce6 and Zn^2+^ ions, Ce6 could be easily loaded onto ZIF-8 by one-pot encapsulation (Fu et al., 2020). Thus, ZIF-8 is usually applied in the research of cancer therapy.

SDT has the following advantages: greater safety, ease of operation, high precision, non-invasiveness, strong penetration into the deep tumor, and fewer side effects (Pan et al., 2018). In particular, because of its strong penetration into deep tumors, it could be applied for deep tumor therapy, i.e., liver and pancreatic tumors. Hence, it has been widely used in cancer therapeutic research. SDT takes the combination of O_2_, low-intensity ultrasound (US), and sonosensitizers (all non-toxic) to generate lots of ROS that can kill tumor cells (Gushchina et al., 2015; An et al., 2020a; Son et al., 2020). A high concentration of ROS can kill tumor cells since it cannot be immediately cleared by the cells and its over-accumulation induces hypoxia and oxidative damage, leading to apoptosis, autophagy, or pyroptosis of cells (Zhou et al., 2016; Rengeng et al., 2017; Yu et al., 2019). However, a lot of O_2_ is required for SDT to play the anti-tumor function, and since most solid tumors have a hypoxic environment, it limits the anti-tumor efficacy. This problem can be solved by two strategies. The first one is by utilizing the nanoparticles to carry O_2_ and help in killing tumor cells. The second one is by using nanoparticles to encapsulate chemotherapeutic drugs that are activated by hypoxia. After stimulating Ce6 by the US, ROS is produced, which consumes O_2_ in tumor cells and creates an extreme oxygen deprivation environment for activating the hypoxia-activating drugs to kill hypoxic cells. Hypoxia-activating drugs could be activated by hypoxia, and produce strong cytotoxicity to cells by releasing transient oxidizing radicals (Li et al., 2017). This achieved a synergistic therapeutic effect with the oxygen-consumed SDT.

**FIGURE 1:**
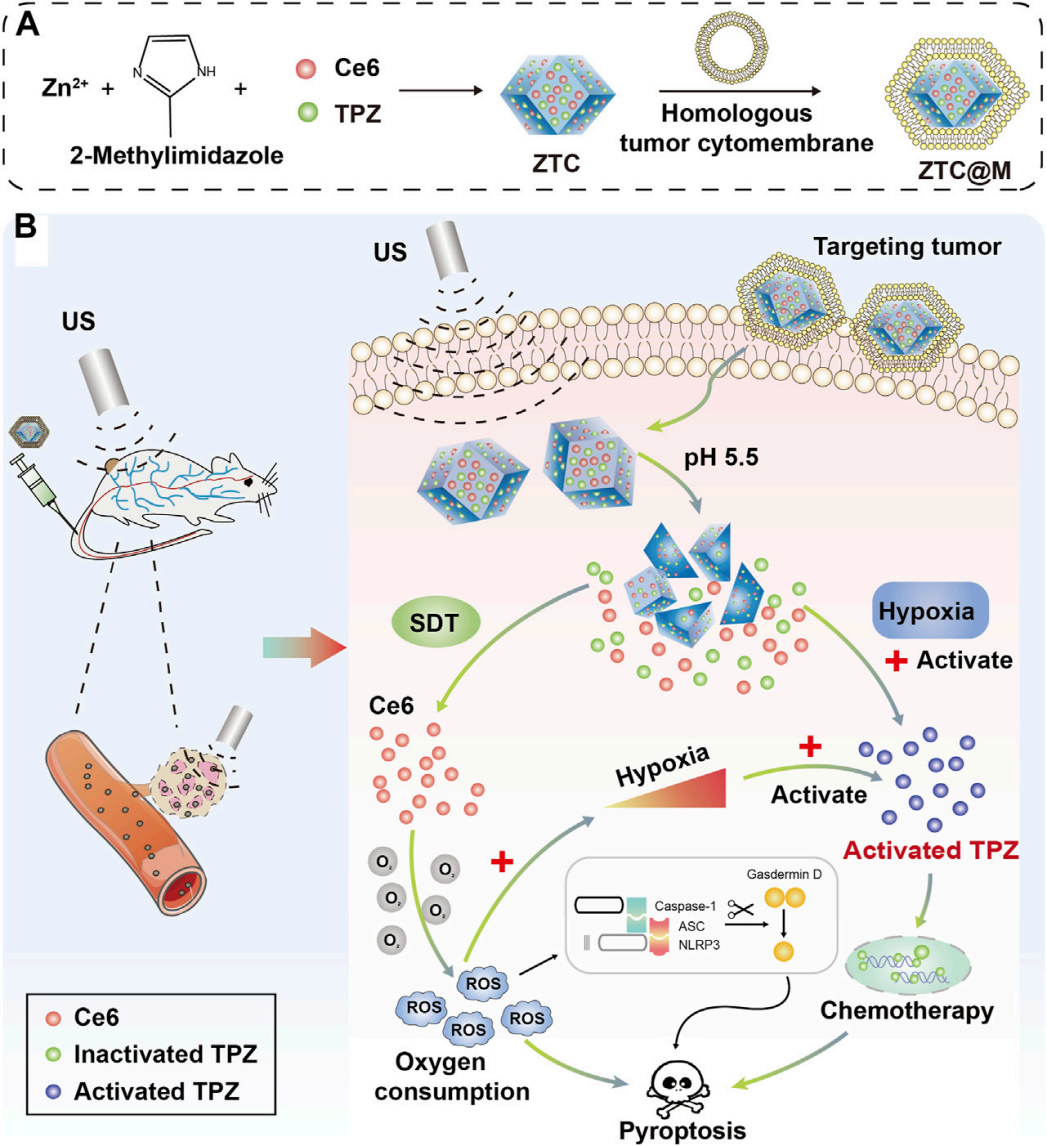
**(A)** Illustration of the synthetic procedure for ZTC@M nanoparticles. **(B)** Schematic diagram of the therapeutic mechanism of pH-sensitive US-triggered hypoxia-activated SDT combined chemo-therapy.

In the study, we aimed to synthesize a nanoparticle of ZIF-8 loading Ce6 and TPZ, and wrap it with homologous tumor cytomembrane to enhance its targeting ability. Irradiated with the US, activated Ce6 could produce ROS to kill tumor cells and further aggravate hypoxia of the tumor microenvironment. Then, TPZ could be greatly triggered and enhance its cell-killing effect (Figure 1). Therefore, our work might provide a potential method for the potent cooperation of SDT and chemotherapy for cancer therapy.

## Materials and Methods

### Materials

Zinc nitrate hexahydrate (Zn(NO_3_)_2_·6H_2_O), 1,2-dimethylimidazole (1,2-MIL), and 1,3-diphenylisobenzofuran (DPBF) were purchased from Aladdin (China). TPZ was purchased from MCE (United States), while Ce6 was purchased from Frontier Scientific (United States). The primary antibodies against GAPDH, ASC, and NLRP3 were purchased from Proteintech (China). Anti-cleaved-caspase-1 and anti-hypoxia-inducible factor 1-alpha (HIF-1α) were purchased from CST (United States). Anti-GSDMD-N was purchased from Abcam (United States).

### Synthesis of ZIF-8@TPZ/Ce6 (ZTC)

Solutions A and B were prepared by dissolving 300 mg of Zn(NO_3_)_2_·6H_2_O (A) and 170 mg of 1,2-MIL (B) in methanol (40 ml) separately. Then, solution B was added to A and stirred thoroughly in a flask for 2 min. Next, TPZ and Ce6 (1 mg/200 µl) methanol solutions were added to the solution and stirred continuously for 5 min. After allowing it to stand for 4 h, the solution was centrifuged at 12,000 rpm for 10 min, followed by two steps of washing with methanol and DMSO. This solution was then freeze-dried. Similarly, ZIF-8@Ce6 (ZC) and ZIF-8@TPZ (ZT) were synthesized following the same procedure. The loading efficiency of Ce6/TPZ was calculated as follows:

$$\frac{Total\;quality-Quality\;in\;the\;supernatant}{Total\;quality\;}\times 100\text{\%}$$

### Synthesis of ZIF-8@TPZ/Ce6@cytomembrane (ZTC@M)

The cytomembrane of the AGS cell was collected using the Membrane Protein Extraction Kit (Fudebio-tech, China). Then, ZTC (1 ml, 2 mg/ml) was added to the cytomembrane fragment of AGS (1 ml, 4 mg/ml) and stirred at 4°C for 2 h. After repeatedly using a liposome extruder (membrane of polycarbonate porous, 400 nm), the spare cytomembrane fragments were discarded *via* centrifugation (4°C, 12,000 *g*, 10 min). Finally, the ZIF-8@Ce6/TPZ@cytomembrane (ZTC@M) was acquired. Following the same procedure, we obtained the cytomembrane of breast cancer 4T1 cell and synthesized ZTC@ cytomembrane 4T1 (ZTC@m).

### Gel Electrophoresis

The AGS cytomembrane and ZTC@M with the same concentration were loaded onto the 10% SDS-PAGE (sodium dodecyl sulfate-polyacrylamide gels) for separation. Coomassie Blue (Beyotime, China) was used to stain the gel, and the picture was taken after destaining the gel with water for 6 h.

### *In vitro* Drug Release at Different pH

To verify the pH sensitivity of ZTC@M, the drug release in PBS at pH 5.5/7.4 was conducted. ZTC@M (2 mg/ml) was dissolved in PBS at pH 5.5 and 7.4, respectively, and put into a constant temperature shaker at 37°C for 6 h. The solution (500 µl) was collected and centrifuged (12,000 rpm, 10 min) at seven time points (0, 1, 2, 3, 4, 5, and 6 h). Equal dosages of PBS were replenished. The absorbance of TPZ/Ce6 in the supernatant was determined at 470 nm (TPZ) and 660 nm (Ce6), respectively. Each group was designed to have three parallel samples. The release rate of TPZ/Ce6 was calculated as follows:

$$Release\;rate\;of\;TPZ/Ce6\left(\%\right)=\;\frac{Quality\;of\;released\;}{Total\;quality\;of\;TPZ/Ce6}\times 100\text{\%}$$

### Production of Reactive Oxygen Species

The production of ROS by ZTC@M was determined by 1,3-diphenylisobenzofuran (DPBF). The absorbance values of mixture DPBF and ZTC@M were measured using the US at 410 nm per minute. The other two groups, i.e., the DPBF and DPBF + ZTC@M groups, were set as the control groups. The remaining DPBF was calculated as follows:

$$Remaining\;DPBF\;\left(\%\right)=100-\frac{OD_{Intial}-OD_{Final}}{OD_{intinal}}\times 100\%$$

### Cytotoxicity Assay

CCK-8 assay was performed to determine the cytotoxicity against the AGS cells. AGS cells were seeded in 96-well plates and incubated overnight. Then, different concentrations of ZTC@M were added to each well and cultured for 24 h. Each group had five parallel samples. After washing with PBS, 100 µl of the medium along with 10 µl of CCK-8 solution was added to each well. After incubating for 1 h, the OD was detected at 450 nm by a microplate reader, and the cell survival rate was calculated.

### Hemolysis Assay

The hemolysis reaction was used to analyze the blood compatibility of nanoparticles. The fresh blood was collected from mice and washed repeatedly with PBS to obtain red blood cells (RBC). Different concentrations of nanoparticles with a constant volume of 500 µl of PBS were added to 500 µl of RBC solution. Each group was designed with three parallel samples, where deionized water and PBS were positive control (PC) and negative control (NC), respectively. After further incubation at 37°C for 4 h, all samples were centrifuged. The absorption of the supernatant was determined at 540 nm by the VARIOSKAN LUX microplate reader. The hemolysis rate was calculated:

$$Hemolysis\;rate\;\left(\%\right)=-\frac{OD_{text}-OD_{NC}}{OD_{PC}-OD_{NC}}\times 100\%$$

### Cellular Uptake

AGS cells (1×10^5^/well) were seeded in a confocal dish and incubated overnight. The confocal dishes were seeded with medium containing ZTC@M (TPZ 12 μg/ml and Ce6 2 μg/ml), which was cultured for 4 h at pH 7.4 in the dark. The cells were then washed with PBS thrice and stained with DAPI and imaged using laser confocal scanning microscopy (LCSM).

Additionally, to analyze the uptake of ZTC@M in different groups, the FACSVerse flow cytometry (BD, United States) was used to analyze the Ce6 fluorescence intensity (FL) of AGS cells.

### *In vitro* Detection of ROS

2,7-Dichlorodihydrofluorescein diacetate (DCFH-DA, Beyotime, China) was applied to determine the ROS level. AGS cells were seeded into a 6-well plate and cultured for 24 h. Then, the mediums containing different nanoparticles (ZC, ZT, ZTC, ZTC@M, and ZTC@M + US) (TPZ 12 μg/ml and Ce6 2 μg/ml) were added to cultured cells and incubated for 4 h in the dark. The US was applied at 1.0 MHz, 1.5 W/cm^2^ for 3 min. The medium was then removed and washed thrice using PBS, and then the cells were incubated with DCFH-DA for 30 min. The green fluorescence imaging of ROS in AGS cells was obtained using fluorescence microscopy.

### *In vitro* detection of hypoxia and immunofluorescence

Image-iT™ Green Hypoxia Reagent was used to detect the oxygen concentration *in vitro*. AGS cells were seeded into the confocal dishes and incubated for 24 h. After incubating the cells with different nanoparticles for 4 h and irradiating with the US (1.0 MHz, 1.5 W/cm^2^, 3 min), cells were washed thrice with PBS. Then, Image-iT™ Green Hypoxia Reagent stock solution (2.5 µM) was added to the cells, and then incubated for 30 min. The cells were then stained with DAPI for 10 min and imaged using LCSM.

The immunofluorescence of HIF-1α was conducted to value the hypoxia of cells. Cells were inoculated on the coverslip. After treatment with nanoparticles, cells were fixed with 4% paraformaldehyde for 15 min. Successively, 0.1% Triton X-100 and 10% BSA were used to permeabilize and block cells. The primary antibody HIF-1α (1:500) was added to cells and incubated overnight at 4°C. Subsequently, cells were incubated with secondary antibody (1:500, Beyotime, China) for 30 min. Finally, DAPI was used to label the nuclei, and the images were taken by an inverted microscope.

### Cell Apoptosis

The flow cytometry assay was used to evaluate the apoptosis of AGS cells. AGS cells (3×10^5^ cells/well) were seeded into a 6-well plate and incubated for 24 h. After washing the cells thrice with PBS, the medium containing ZC, ZT, ZTC, ZTC@M, and ZTC@M + US (TPZ 12 μg/ml and Ce6 2 μg/ml) was added to the plates, respectively, and cultured for 4 h. The US was applied at 1.0 MHz, 1.5 W/cm^2^ for 3 min. Next, supernatants and cells were collected and then were mixed with 500 µl of binding buffer, to which 5 µl of Annexin V-FITC and 10 µl of PI were added and incubated for 5 min in the dark. Finally, cell apoptosis was detected using FACSVerse flow cytometry (BD, United States).

### Live/Dead Cell Staining

The Calcein/PI Live/Dead Viability/Cytotoxicity Assay Kit (Beyotime, China) was applied to analyze the live/dead cell staining of AGS. The AGS cells (3×10^5^ cells/well) were seeded into a 6-well plate and incubated for 24 h. After washing the cells thrice with PBS, the medium containing ZC, ZT, ZTC, ZTC@M, and ZTC@M + US (TPZ 12 μg/ml and Ce6 2 μg/ml) was added to the plates, respectively. The US was applied at 1.0 MHz, 1.5 W/cm^2^ for 3 min. After culturing the cells for 4 h, 1 ml of detecting buffer was added to the cells along with 1 µl of Calcein and 1 µl of PI and incubated for 30 min to stain the live and dead cells. The fluorescence of AGS cells was observed under a fluorescence microscope.

### *In vivo* Safety Experiment

The female nude mice (5 weeks) were randomly divided into three groups. Two groups of mice were injected with 200 µl of ZTC@M (10 mg/kg Ce6 and 40 mg/kg TPZ) every 3 days for 3 times through the tail vein. The other group was used as the control, which was injected with an equal amount of PBS. The mice were sacrificed after 7 days and 30 days, respectively. Later, mice blood was collected and analyzed. Also, organs (heart, liver, spleen, lung, and kidney) were collected and stained for hematoxylin and eosin (H&E).

### *In vivo* Therapy

Strictly, all the animal experiments were conducted under the guidelines of the National Animal Management Regulations of China and were approved by the Animal Experimental Ethics Committee of Guangxi Medical University.

The nude mice bearing AGS tumors were used to investigate the inhibition effect of ZTC@M + US on tumor growth. A total of 3×10^5^ AGS cells were injected into nude mice. When the tumors grew up to 80–100 mm^3^, the AGS tumor-bearing nude mice were randomly divided into six groups (*n* = 5): (1) PBS, (2) PBS + ZC, (3) PBS + ZT, (4) PBS + ZTC, (5) PBS + ZTC@M, and (6) PBS + ZTC@M + US. PBS containing different nanoparticles (200 μl, 10 mg/kg Ce6, and 40 mg/kg TPZ) were injected into the mice *via* tail vein every 3 days for 3 times, and US (1.5 W/cm^2^, 3 min) was used to irradiate group 6 after 6 h of injection. The mice were observed for 20 days. The body weight and tumor volume were recorded every 2 days. All mice were sacrificed at the end of observation, and the tumors were collected followed by H&E staining, TUNEL staining, and immunohistochemistry (IHC) staining. Tumor volume and tumor inhibition rate (TIR) were calculated.

$$Tumor\;Volume\;\left(cm^{3}\right)=Tumor\;length\;\times Tumor\;width^{2}/2$$

$$TIR\left(\text{\%}\right)=\left(V_{CON}-V\right)/V_{CON}\times 100\%$$

### Western Blot

AGS cells (3×10^5^ cells/well) were seeded into a 6-well plate. Once the cells reached approximately 90% confluence, the medium containing ZTC@M (TPZ 12 μg/ml and Ce6 2 μg/ml) was added to the plates and cultured for 24 h. The US was applied at 1.0 MHz, 1.5 W/cm^2^ for 3 min. Next, the cells were collected and lysed with RIPA buffer with PMSF. All protein samples were loaded onto the 10% SDS-PAGE and then transferred onto a PVDF (polyvinylidene fluoride) membrane. After incubating the membranes with blocking solution for 30 min, the PVDF membranes were incubated with primary antibodies against ASC (1:500), NLRP3 (1:500), HIF-1α (1:1,000), GSDMD-N (1:1,000), cleaved-caspase-1 (1:1,000), mature IL-1β (1:500), and GAPDH (1:10,000) overnight, respectively. The membranes were then washed thrice with TBST and incubated with secondary antibodies. Finally, the results were visualized using the Pierce ECL Western Blotting Substrate.

### Statistical Analysis

All data were presented as the mean ± standard deviation (SD). Descriptive statistics and ANOVA (single-factor analysis of variance) were used to analyze data. The Student’s *t*-test was used to analyze the difference between the two groups. Prism 8 software (GraphPad 8) was used for all the statistical analyses. *p* < 0.05 was considered statistically significant.

## Results and Discussion

### Characteristic of Nanoparticles

The morphology and structure of ZTC@M nanocomposites were characterized using scanning electron microscopy (SEM, SU8020, Japan) and transmission electron microscopy (TEM, JEM-2100F, United States). The size and surface potential of nanomedicines were detected by ZS90 Zeta Sizer.

**FIGURE 2:**
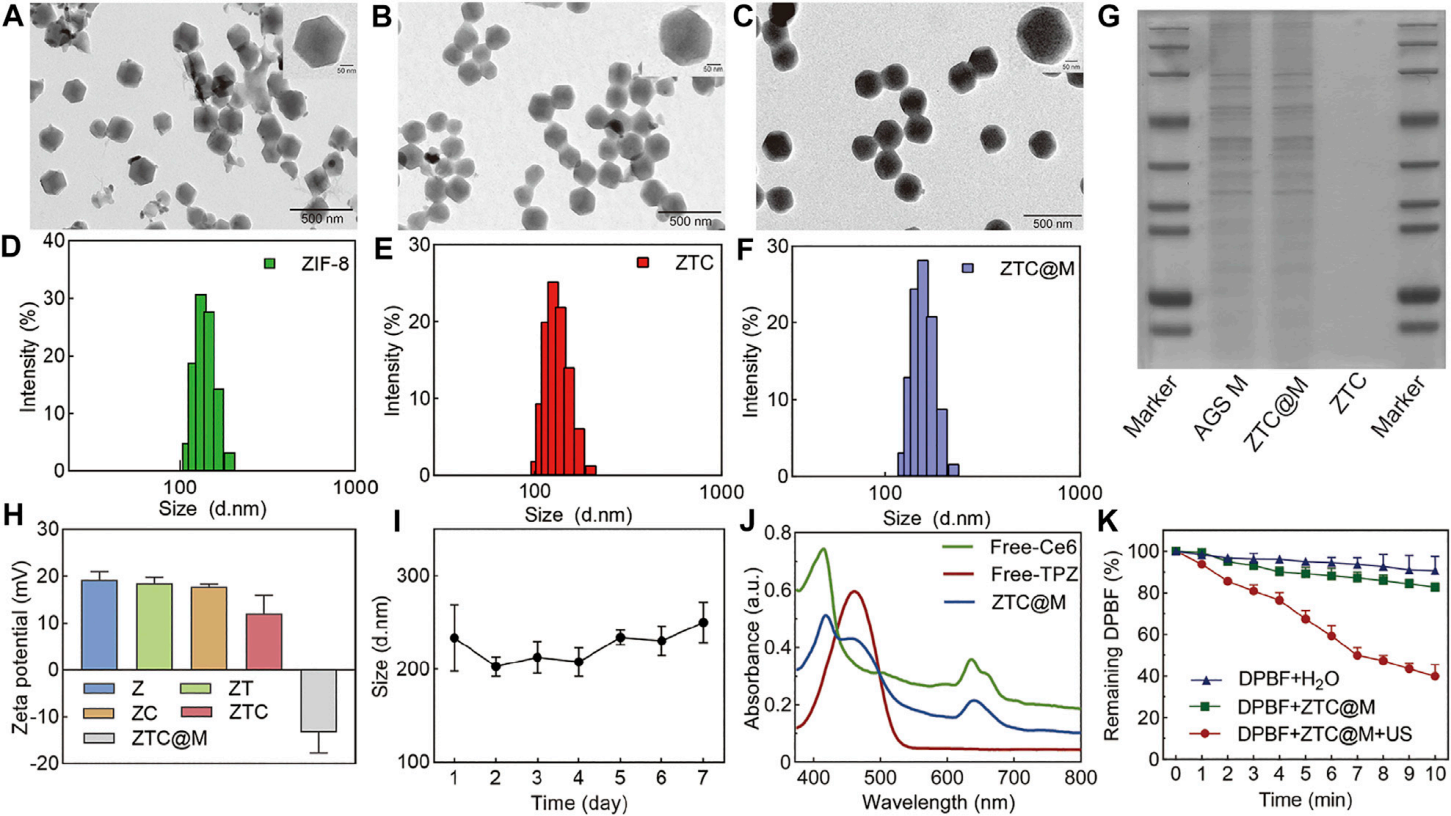
Characterization of ZTC@M nanoparticles. TEM and magnification images of ZIF-8 **(A)**, ZTC **(B)**, and ZTC@M **(C)**. The scale bar is 200 nm. Hydrated nanoparticle size distribution of ZIF-8 **(D)**, ZTC **(E)**, and ZTC@M **(F)**. Gel electrophoresis of AGS cytomembrane and ZTC@M **(G)**. Zeta potential of different nanoparticles **(H)**. The change of hydrated nanoparticle size of ZTC@M **(I)**. The ultraviolet-visible absorption spectra of free-Ce6, free-TPZ, and ZTC@M **(J)**. Reactive oxygen species (ROS) production by ZTC@M under different conditions **(K)**.

ZTC was prepared using zeolitic imidazolate framework 8 (ZIF-8) coloaded with TPZ and Ce6. The final nanoparticle called ZTC@M was prepared by wrapping cytomembrane fragments of AGS cells. TEM images showed that ZTC had the same structure as ZIF-8 (Figures 2A,B) with a uniform size (Figures 2D,E). The ZTC@M nanoparticle was covered with cytomembrane as seen through the TEM (Figure 2C), and the average thickness of cell membrane on the surface of ZTC@M was about 20 nm. The size was also bigger than ZTC (Figures 2E,F), with an increase in size reported from 199 to 253 nm. Besides, the comparison with the surface potential before modification of AGS cytomembrane showed a reversal from positive (13.8 mV) to negative (–16.4 mV), indicating successful coating of AGS cytomembrane (Figure 2H). This result was consistent with the gel electrophoresis result (Figure 2G). Also, the stability of ZTC@M was evaluated, where the results showed that the size did not change in ultrapure water within a week (Figure 2I), indicating that the nanoparticle was stabilized.

The absorption peaks of TPZ (470 nm) and Ce6 (400 and 660 nm) were presented in the absorption spectrum of ZTC@M (Figure 2J), indicating successful loading of the nanoparticle with TPZ and Ce6. Simultaneously, the loading efficiencies of Ce6 and TPZ were calculated, which were found to be 77.57 ± 0.48% and 53.86 ± 3.48%, respectively. The results illustrated that the frame ZIF-8 could co-load both TPZ and Ce6 for the cooperative therapy.

DPBF is a typical analytical reagent of ROS and is used to analyze the production of ROS. Compared to the control groups, the remaining DPBF in the ZTC@M group irradiated with the US was found to be significantly lower (Figure 2K), indicating that ZTC@M upon US irradiation could produce much more ROS from Ce6.

**FIGURE 3:**
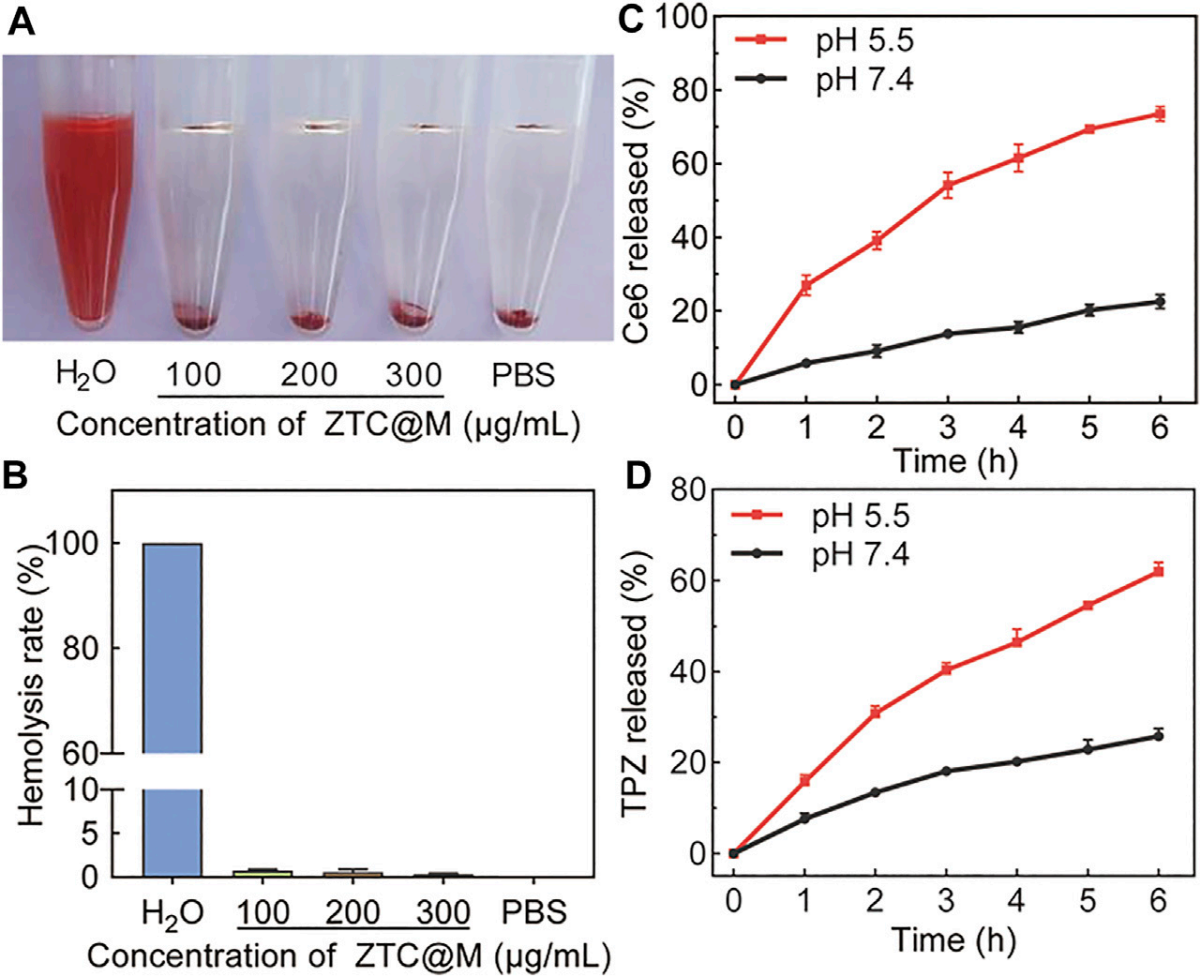
Hemolysis assay of ZTC@M at different concentrations **(A)** and hemolysis ratio of red blood **(B)**. Ce6 **(C)** and TPZ **(D)** controlled release curves of ZTC@M at different pH.

The hemolysis assay was conducted to analyze the biocompatibility of ZTC@M. The hemolysis rate below 10% was considered safe for intravenous injection (Xu et al., 2015). The hemolysis rate was observed to be less than 1% when the concentration of ZTC@M used was up to 300 μg/ml (Figures 3A,B), indicating that the nanoparticle had good biocompatibility.

The drug-releasing ability of TPZ and Ce6 from ZTC@M was evaluated in PBS at two different pH. Because of the pH-responsive property, it was observed that the accumulative release rate of Ce6/TPZ was higher at lower pH (Figures 3C,D). Incubation for 6 h at pH 5.5 released approximately 61.95 ± 1.40% of loaded TPZ and 73.57 ± 2.01% of loaded Ce6 from the ZTC@M, and these values were much higher than those obtained at pH 7.4 (Figures 3C,D), demonstrating that tumor acidic microenvironment was helpful to the release of Ce6/TPZ. Thus, the pH responsiveness of ZTC@M was applied to release drugs in an acidic solution to attain a combined target treatment.

### Cellular Uptake

**FIGURE 4:**
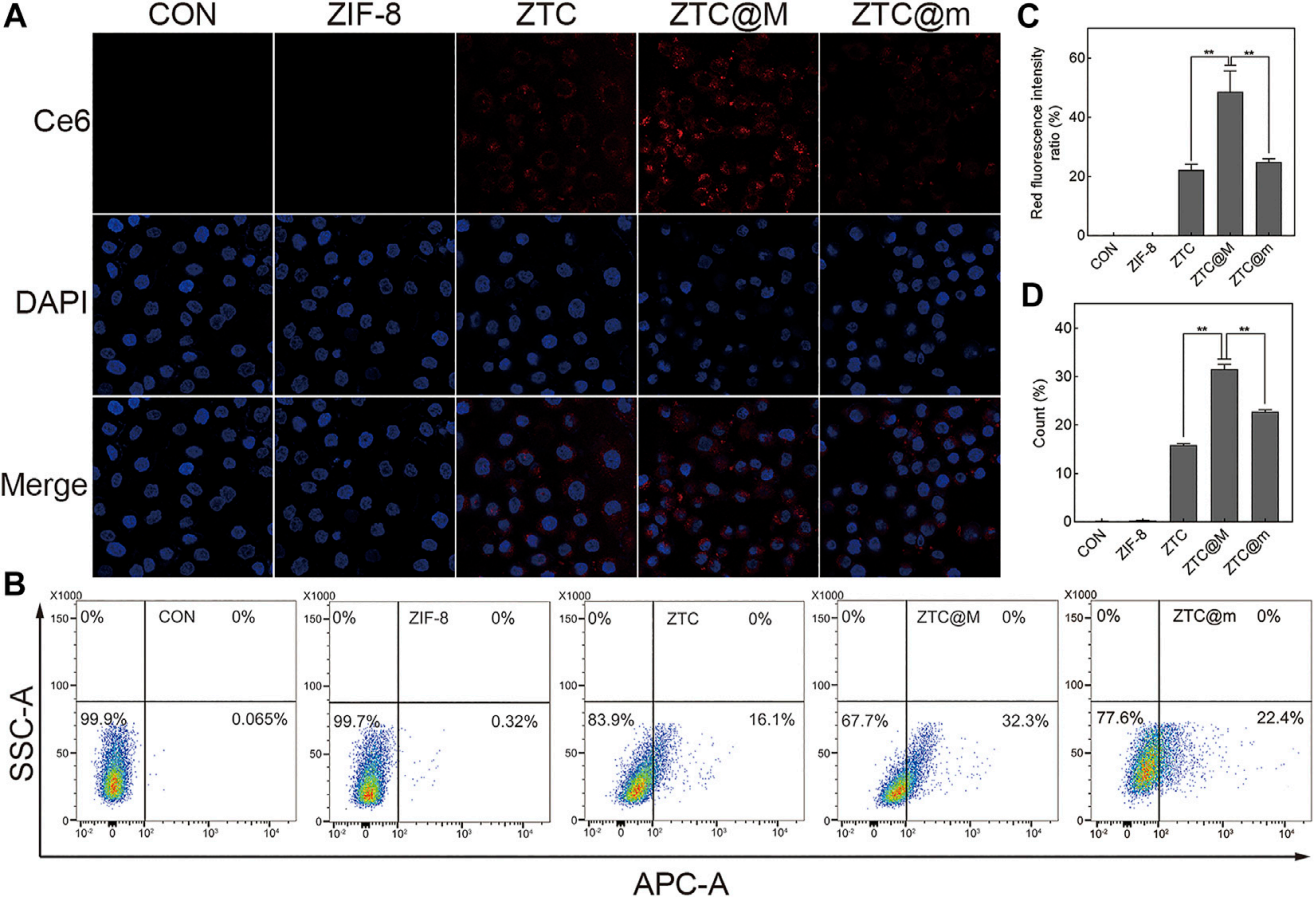
Cellular uptake experiments. Fluorescence imaging **(A)** and flow cytometry **(B)** of AGS cells incubated with different nanoparticles (600×). Quantification of intracellular Ce6 fluorescence intensity in **A (C)**. Count of AGS cells with intracellular fluorescence of **B (D)**. *p < 0.05, **p < 0.0001.

To evaluate the targeting ability of nanoparticles, ZTC nanoparticles were wrapped with different tumor cytomembranes. ZTC@M was ZTC nanoparticle modified with homologous tumor cytomembrane of AGS cells, and ZTC@m was ZTC nanoparticle modified with cytomembrane of breast cancer 4T1 cells. After the addition of cytomembrane of AGS, stronger fluorescence intensities were presented in the ZTC@M group, comparing with the ZTC group (*p* < 0.0001) (Figures 4A,C). Furthermore, the cell signal intensities treated with ZTC@m showed weaker signals than that in the ZTC@M group (*p* < 0.0001) (Figures 4A,C). The results of the flow cell experiments agreed with the results of the cell fluorescence (Figures 4B,D). Because of the homologous cytomembrane, more nanoparticles can be taken up by the cells. These results indicated that ZTC@M could be recognized better by AGS cells and made use of the tumor acidic microenvironment to target release the drug and achieve anti-tumor function.

### *In vitro* ROS/Hypoxia Assay

**FIGURE 5:**
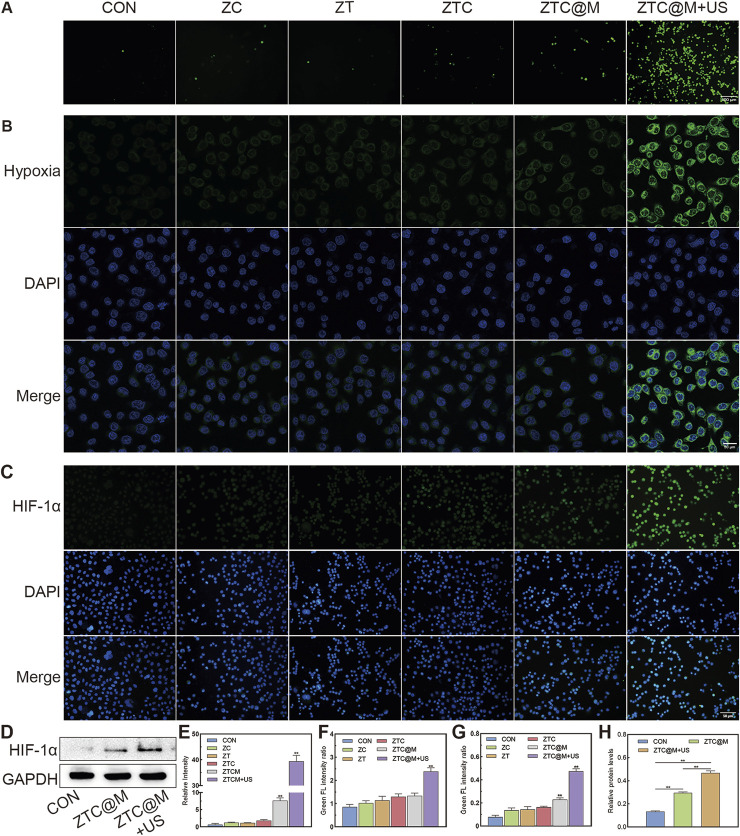
*In vitro* ROS/hypoxia assay and IF. ROS production was detected by fluorescence of DCFH-DA in AGS cells **(A)** (100×). Intracellular hypoxia imaging using Image-iT™ Green Hypoxia Reagent as the syndicator **(B)** (200×). Immunofluorescence images of AGS stained by HIF-1α **(C)** (200×). Gel images of HIF-1α in AGS cells treated with ZTC@M + US **(D)**. Quantification of intracellular fluorescence intensity in A **(E)**, B **(F)**, and C **(G)**. The bar chart indicates the relative density of HIF-1α to GAPDH in D **(H)**. *p < 0.05, **p < 0.0001.

Intracellular ROS levels were evaluated by DCFH-DA, whose green fluorescence was enhanced with the increase in ROS level inside the cells. As presented in Figures 5A,E, the intensity of ROS green fluorescence in the group irradiated by the US (ZTC@M + US) was found to be much higher than that found in ZC, ZTC, and ZTC@M groups without the US (*p* < 0.0001), which indicated that Ce6 irradiated by the US released ROS. The reason behind this could be attributed to Ce6 being a sonosensitizer, which may produce ROS after the treatment with the US.

The continuous oxygen consumption of ROS during the treatment would aggravate hypoxia of tumor microenvironment (Pingar et al., 1992; Li et al., 2017). This could create a convenient condition for the activation of TPZ. Then, the ability to induce hypoxia by ROS was evaluated by the hypoxia reagent. As the end-point assay reagent, the signal of Image-iT™ Green Hypoxia Reagent increases with the reduction of oxygen levels. The green fluorescence is enhanced with the increase in hypoxia level. Our results showed that the green fluorescence in the CON group was weaker than that of the ZTC (*p* = 0.0068), ZTC@M (*p* = 0.0034), and ZTC@M + US (*p* < 0.0001) groups (Figures 5B,F). Meanwhile, the strongest green fluorescence was seen in the ZTC@M + US group, which was even stronger than that of the ZTC@M group (*p* < 0.0001) (Figures 5B,F).

HIF-1α is considered a key mediator of signal in poorly oxygenated areas and can stay steady under a hypoxia environment (Robertson et al., 2014; Harrison et al., 2018). In normoxia conditions, HIF-1α is always inactivated and degraded so that it is hardly detected (Masoud and Li, 2015). HIF-1α can stay stabilized in poor oxygen conditions and can be translocated to the nucleus responding rapidly to oxygen deprivation (Ke and Costa, 2006). Furthermore, IF and WB of HIF-1α were performed to demonstrate the aggravation of intracellular hypoxia. It was found that the observed green fluorescence in the ZTC@M + US group was stronger than that in any other group, i.e., CON, ZC, ZT, ZTC, and ZTC@M groups (*p* < 0.0001) (Figures 5C,G), showing that the hypoxia level of the ZTC@M + US group was the strongest. In Figures 5D,H, the levels of HIF-1α protein in the ZTC@M + US group were found to be much higher than that of the CON group (*p* < 0.0001) and the ZTC@M + US group (*p* < 0.05), which was in accord with the results of IF. These results proved that the ROS released from Ce6 could enhance intracellular hypoxia, which provided a hypoxic environment to TPZ.

### *In vitro* Therapeutic Effect

**FIGURE 6:**
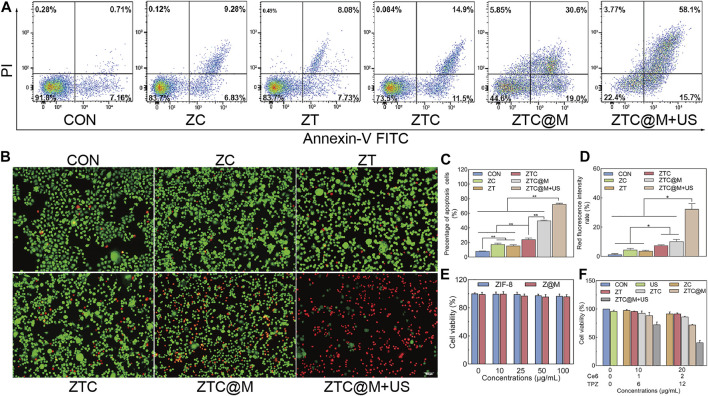
*In vitro* therapeutic effect. Calcein-AM and PI staining fluorescence images of AGS cells after various treatments **(A)** (100×). Flow cytometry analysis for apoptosis of AGS cells after various treatments **(B)**. Quantification of intracellular fluorescence intensity in **A (C)**. The bar chart indicates the percentage of apoptosis cells in **B (D)**. Cell viabilities of AGS cells treated with various probes at different concentrations in **(E)** and **(F)**. *p < 0.05, **p < 0.0001.

To evaluate the killing effect of nanoparticles, CCK-8 assay, apoptosis assay, and Calcein/PI staining were conducted. The apoptosis assay presented that the percentages of apoptotic cells in CON, ZC, ZT, ZTC, ZTC@M, and ZTC@M + US groups were approximately 8.13%, 17.40%, 15.49%, 24.10%, 49.87%, and 72.77%, respectively (Figures 6A,C). There was a significant difference observed between ZTC and ZT/ZC. A higher rate of apoptosis was observed in ZTC@M compared to that of the ZTC. The highest apoptosis rate was demonstrated in the group treated with US (ZTC@M + US).

Similar results were shown by Calcein-AM/PI staining experiments. Figures 6B,D show that the red fluorescent intensities from dead cells in the ZTC group were stronger than those found in the ZT and ZC groups (*p* < 0.05), indicating that the efficiency of nanoparticles was better in combination with Ce6 and TPZ than that of a single drug. Meanwhile, the red fluorescence intensities of the cytomembrane target group (ZTC@M) were higher than those groups without cytomembrane (ZTC) (*p* < 0.05). This was because the homologous AGS cytomembrane specifically increased the uptake of nanoparticles by AGS cells and enhanced the killing effect on tumor cells. Also, after the US treatment, a much stronger red fluorescence was seen in most of the cells. The quantitative analysis of fluorescence presented that the therapeutic effect of the SDT treatment group (ZTC@M + US) was found to be higher than that of the group without the SDT (ZTC@M) (Figures 6B,D) (*p* < 0.05).

ZIF-8 and ZIF-8@Cytomembrane (Z@M) were nearly non-toxic towards AGS cells, whose viabilities were still above 90% when the probe concentrations reached 100 μg/ml (Figure 6E). Besides, treatment with the US alone was nearly non-toxic towards AGS cells as well (Figure 6F). The cell viabilities treated with different nanoparticles at a concentration of 20 μg/ml (Ce6 2 μg/ml equivalent, TPZ 12 μg/ml equivalent) were lower than those treated at 10 μg/ml (Ce6 1 μg/ml equivalent, TPZ 6 μg/ml equivalent), suggesting that the cytotoxicity of nanoparticles was strengthened with the increase of concentration (Figure 6F). The cytotoxicity of ZTC was found to be stronger than ZT/ZC (Figure 6F), suggesting a strengthened killing effect of a combined drug than a single drug loaded on ZIF-8. The survival rates of AGS cells with ZTC@M were lower than those in the ZTC group (Figure 6F), which meant a stronger killing effect after wrapping with cell cytomembrane. The strongest cytotoxicity was observed in ZTC@M + US (Ce6 2 μg/ml equivalent, TPZ 12 μg/ml equivalent), and the cell viability was inhibited down to approximately 40%, showing that the killing effect was enhanced by SDT.

All these results illustrated that the combination of chemotherapy and SDT could enhance the killing effect on AGS cells.

### *In vivo* Biocompatibility Research

**FIGURE 7:**
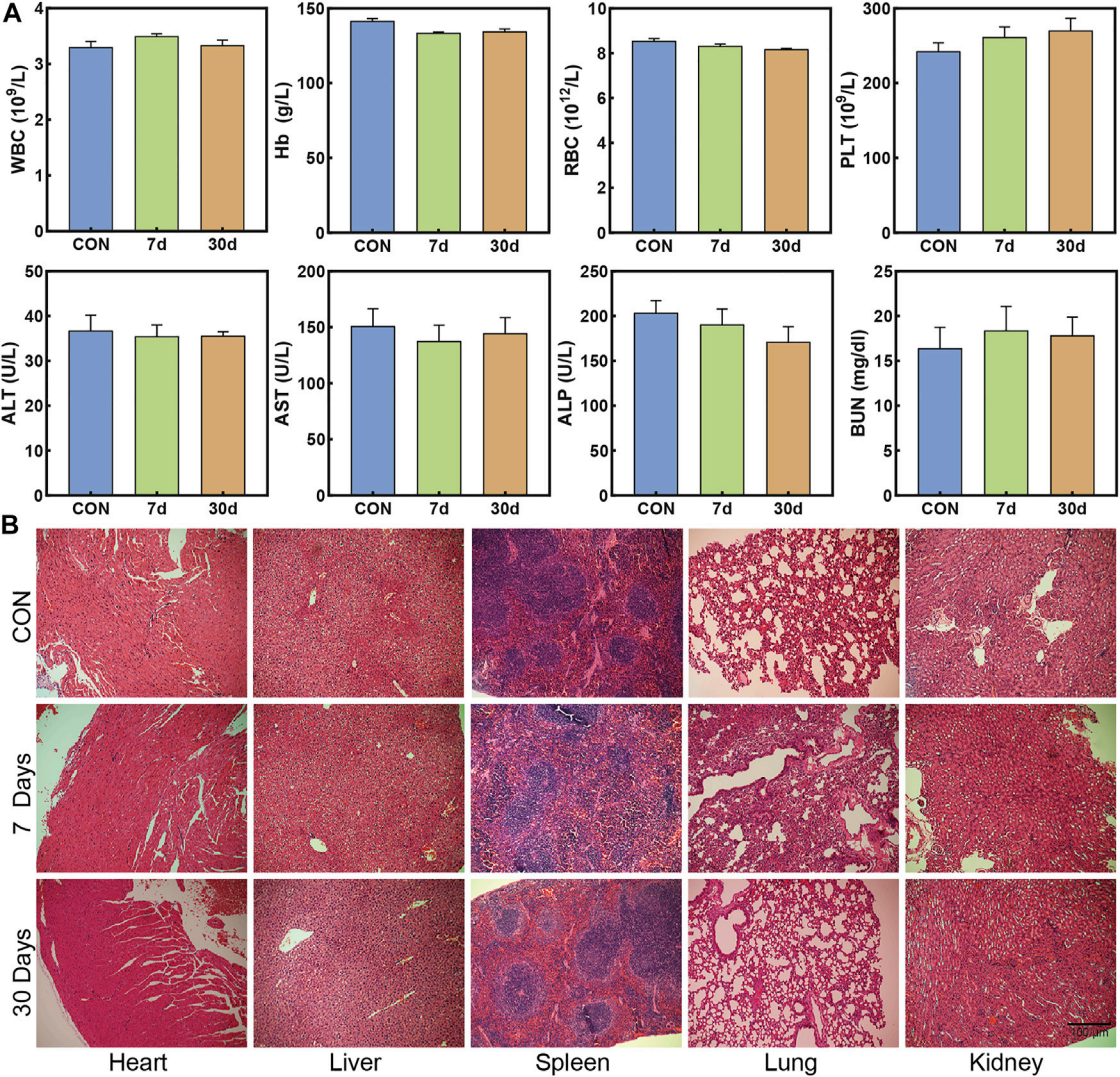
*In vivo* biocompatibility research. The count changes of WBC, RBC, Hb, PLT, ALT, AST, ALP, and BUN **(A)**. H&E staining of nude mice after 7 days and 30 days of three injections of ZTC@M **(B)** (100×).

To verify the safety *in vivo*, we evaluated the biocompatibility of nanoparticles. Figure 7A shows that there were no significant differences in the counts of white blood cell (WBC), red blood cell (RBC), hemoglobin (Hb), and platelet (PLT) between the groups observed on 7/30 days and the control group, illustrating that there was no significant blood toxicity found in ZTC@M *in vivo*. Meanwhile, aspartate aminotransferase (AST), alanine aminotransferase (ALT), alkaline phosphatase (ALP), and blood urea nitrogen (BUN) were detected to analyze the presence of any liver and kidney damage. As shown in Figure 7A, no significant differences were observed in these indexes in the three groups. Also, to confirm whether nanoparticles can induce organ damage, the H&E staining of organs was compared among the three groups. The results demonstrated that no apparent damage or pathological changes were observed in the experimental groups, which showed consistency with the control group (Figure 7B). Hence, these results presented that ZTC@M had good biocompatibility with no apparent toxicity *in vivo*, indicating it to be a potential nanoparticle for *in vivo* anti-tumor experiments.

### *In vivo* Combined Chemo-SDT

**FIGURE 8:**
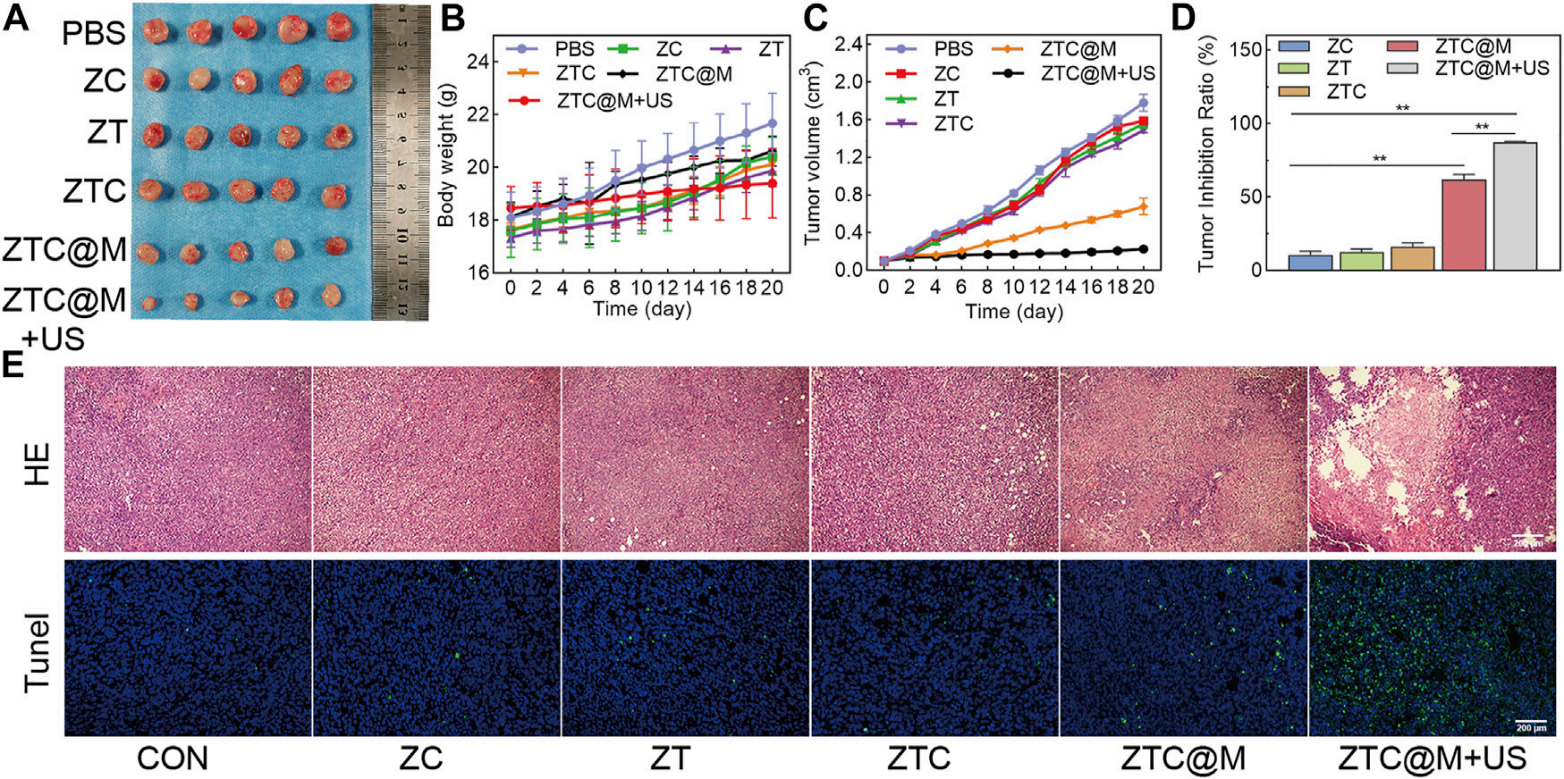
*In vivo* SDT. Photos of tumor **(A)**, tumor volume change **(B)**, body weight change **(C)**, and tumor inhibition rate **(D)** of AGS tumor-bearing nude mice with different treatments. H&E and TUNEL staining of tumor after different treatments **(E)** (100×). *n* = 5. *p < 0.05, **p < 0.0001.

Nude mice bearing AGS tumors were used as the model to investigate the anti-tumor effect of SDT of ZTC@M. As shown in Figure 8B, there was no significant loss of body weight in mice among all six groups during the treatment, which demonstrated that the nanoparticles had good biocompatibility with no evident toxicity to mice. It was observed that the growth of tumors treated with PBS was a little more rapid than those treated with ZC and ZT (Figure 8C), indicating that single-drug therapy had limitations. Simultaneously, Figure 8C shows that the growth of tumors was slightly slower in the group treated with nanoparticles combined with Ce6 and TPZ than that of a single drug, which showed that combined therapy worked relatively better than a single drug that had limited efficiency. However, the growth of the tumor in the targeting group (ZTC@M) was further reduced, and the inhibition of tumor growth was found to be stronger than the previous three groups, because being encapsulated with homologous tumor cytomembrane enhanced the targeting ability of ZTC@M, and it would be recognized more easily by tumor cells. Hence, more nanoparticles could enter tumor cells, which meant more TPZ could be carried to the tumor site. Even without US, the TPZ also could be partly activated because of the local tumor hypoxic environment so that it could kill tumor cells. However, it still could not reduce the growth of the tumor completely. The tumor growth was inhibited in the ZTC@M mice treated with US irradiation compared to those without US irradiation, indicating a significant anti-tumor efficiency by US irradiation. Regarding the tumor inhibition ratio (Figure 8D), the inhibition rate reached up to approximately 87% in the ZTC@M + US group, which was higher than any other group, indicating the same results as the tumor volume. The reasons behind these results might be explained as follows: first, the nanoparticles encapsulated with homologous tumor cytomembrane enhanced its targeting ability and were recognized more easily by tumor cells. Hence, more nanoparticles could enter tumor cells and perform their anti-tumor function. However, without US irradiation, Ce6 could not be activated completely, and the function was limited. Second, after irradiation with the US, the accumulation of ROS released by Ce6 could aggravate hypoxia of the tumor microenvironment and kill tumor cells. Finally, hypoxia further activated the anti-tumor effect of TPZ and induced the death of tumor cells. These results demonstrated that ZTC@M + US could efficiently inhibit tumors and exert anti-tumor function.

Furthermore, H&E staining of the tumor was performed to evaluate the therapeutic efficacy. An obvious necrotic tissue was observed in the tumor tissues treated with the ZTC@M + US group (Figure 8E). Furthermore, the TUNEL staining was performed to observe the tumor injury in mice among the different groups (Figure 8E). The results indicated that there was a small number of apoptotic cells observed in the tumors of PBS, ZC, ZT, and ZTC groups. In the ZTC@M group, many apoptotic cells were seen, but the number was less than that of the ZTC@M + US group. These results were in accord with the results of H&E staining.

Therefore, the above results proved that the ZTC@M + US group could not only inhibit tumor growth, because it could achieve targeting delivery, but also use TPZ to strengthen the effect of SDT, further enhancing co-therapy of chemotherapy and SDT.

### ZTC@M Contributed to AGS Pyroptosis

Some studies reported that ROS contributed to apoptosis and pyroptosis (Zhang et al., 2017; Wang et al., 2019). Pyroptosis is different from cell apoptosis and is an inflammatory form that mediates the programmed death of cells (Teng et al., 2020). It is activated by caspase-1/4/5/11 and can lead to cell damage, including fragmentation of chromatin, cell swelling, lysis of plasma membranes, and release of intracellular proinflammatory contents (Jorgensen and Miao, 2015; Fang et al., 2020). It is well known that caspase-1, NLRP3 (NACHT, LRR, and PYD domain-containing protein 3), and ASC molecules can induce pyroptosis in tumor cells. Caspase-1 is the activator of pyroptosis. ASC comprises a caspase recruitment domain and a pyrin domain and is the adaption protein of NLRP3 (Dong et al., 2019). The NLRP3 inflammasome is the most characterized inflammasome, which consists of the sensory molecule NLRP3, caspase-1, and ASC (Schroder and Tschopp, 2010; Cheng et al., 2020). It is also well known that ROS is an activator of NLRP3 inflammasome (Zhou et al., 2011; Wu et al., 2018). During the forming of NLRP3 inflammasomes, pro-caspase-1 is activated and transformed into cleaved-caspase-1, which can promote interleukin-1 beta (IL-1β) and interleukin-18 (IL-18) to be mature forms (Dong et al., 2019). GSDMD is a member of the gasdermin family, which is crucial for caspase-1-mediated pyroptosis. Due to the inhibitory binding of the GSDMD-N domain and GSDMD-C domain, full-length GSDMD remains inactivated (Shi et al., 2017). However, it can be cleaved by activated caspase-1 (cleaved-caspase-1), which can remove the inhibitory GSDMD-C domain and release the GSDMD-N domain. The released GSDMD-N domain can cause membrane lysis and induce pyroptosis (Liu et al., 2016; Wang et al., 2020). Importantly, the pore-forming ability of GSDMD-N needs the help of IL-1β secretion, they work together to induce cell pyroptosis (Dong et al., 2019).

**FIGURE 9:**
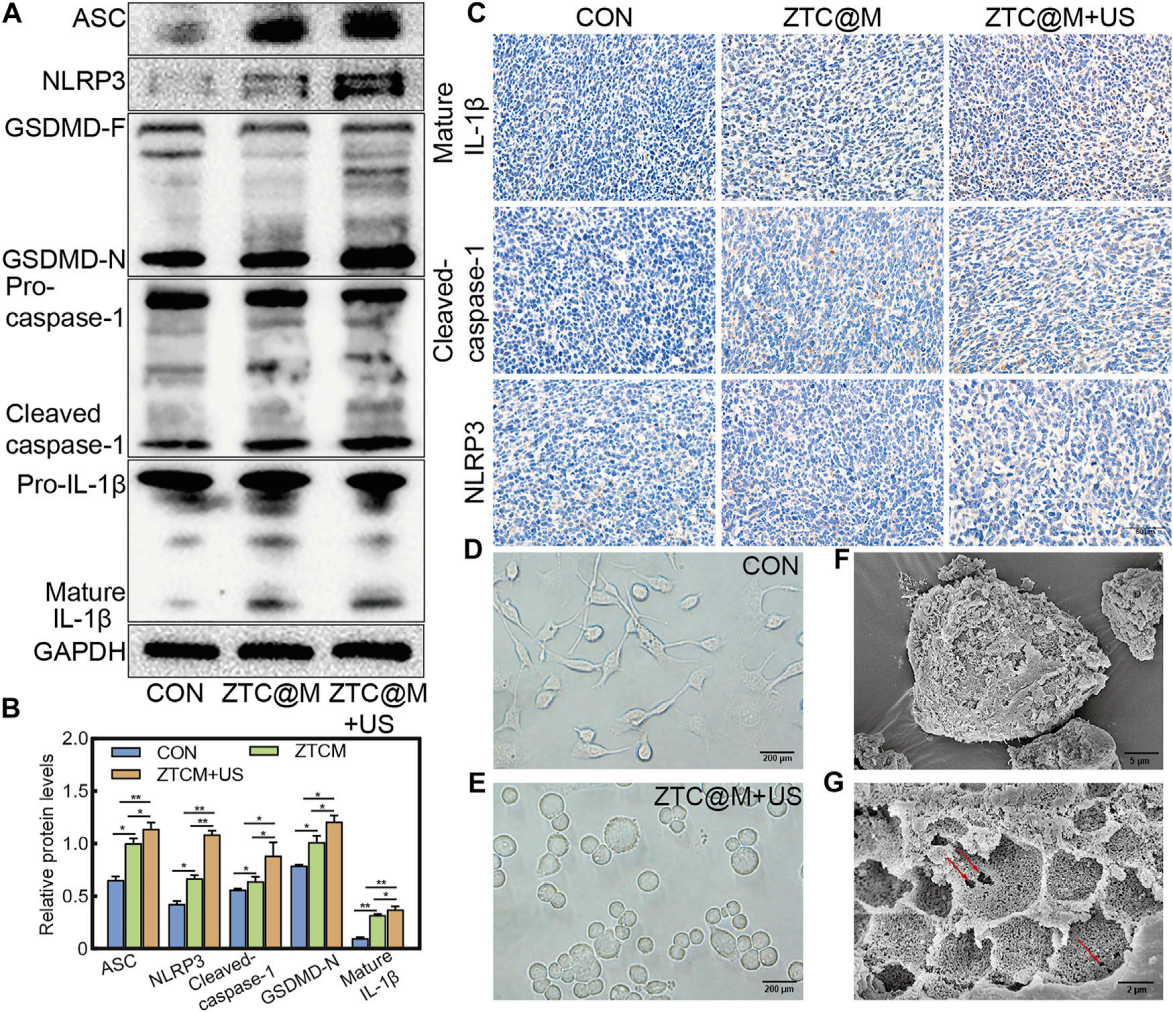
ZTC@M + US activates morphological and molecular characteristics of pyroptosis. The protein levels of cleaved-caspase-1, ASC, GSDMD-N, mature IL-1β, and NLRP3 after SDT **(A)**. The bar chart indicates the relative density of cleaved-caspase-1, ASC, GSDMD-N, mature IL-1β, and NLRP3 to GAPDH in A **(B)**. IHC staining of cleaved-caspase-1, mature IL-1β, and NLRP3 of tumor **(C)**. Phase-contrast photomicrographs showing AGS cells untreated **(D)** and treated with ZTC@M + US **(E)** (100×). Scanning electron micrographs of AGS cells after treatment with ZTC@M + US **(F)** and **(G)**. Scale bars are 5 µm **(E)** and 2 µm **(F)**. *p < 0.05, **p < 0.0001.

To determine if the nanoparticles could induce pyroptosis, the levels of pyroptosis-associated proteins were determined by Western blotting and IHC. The ZTC@M groups exhibited higher levels of cleaved-caspase-1, NLRP3, ASC, mature IL-1β, and GSDMD-N compared to that of the CON group (Figures 9A,B, all *p*-value < 0.05). Compared with the CON and ZTC@M groups, the levels of cleaved-caspase-1, NLRP3, ASC, mature IL-1β, and GSDMD-N to GAPDH in the ZTC@M + US group were found to be further higher (Figures 9A,B, all *p*-value < 0.05). This meant that the NLRP3 inflammasome in AGS cells did activate by ZTC@M + US so it could cleave caspase-1 to be cleaved-caspase-1. After activating, the cleaved-caspase-1 further cleaved GSDMD and IL-1β into GSDMD-N and mature IL-1β, and then induced inflammatory programmed cell death. Furthermore, the IHC results in Figure 9C presented that the expressions of NLRP3, mature IL-1β, and cleaved-caspase-1 were significantly stronger in the ZTC@M + US group than those in CON and ZTC@M groups. These results suggested that the ZTC@M irritation with the US could induce pyroptosis of AGS cells, which enabled it to play the anti-tumor function.

Cells undergoing pyroptosis show distinct morphological features. Deron R. Herr et al. proved that the most prominent pyroptotic cells represented cell swelling, the retraction of cellular processes, and the emerging of pores in the cell surface (Herr et al., 2020). AGS cells were also captured for detailed surface morphology by phase-contrast microscopy (Figures 9D,E) after the ZTC@M + US treatment. Untreated AGS cells appeared normal under microscopy showing characteristics such as extended processes among the cells (Figure 9D). After the treatment with ZTC@M + US, it was found that the loss of processes and cell swelling manifested as a significant increase in the cell size (Figure 9E). Furthermore, the surface morphology was observed by the SEM. The most prominent feature of treated cells was the numerous pits or pores of different sizes across the cell surface (Figures 9F,G); this was correlated with collapsing of the structure and flattening of the cell shape. Simultaneously, it also presented a rounded morphology due to the complete retraction of cellular processes (Figure 9F). This further proved that our nanoparticle could induce AGS cell pyroptosis.

## Conclusion

In summary, we successfully synthesized a US-activated, pH-sensitive, hypoxia-induced ZIF-8 organic-metal framework that targeted the delivery of TPZ and Ce6, which could be used for combining SDT and chemotherapy to enhance the therapeutic effect in GC. Our results showed that the ZTC@M nanoparticle had good biocompatibility, targeting ability, and therapeutic effect. Irradiated with the US, Ce6 could release ROS and kill tumor cells, which played the anti-tumor effect of SDT. The accumulation of ROS aggravated hypoxia of the tumor microenvironment, which could more efficiently activate TPZ to kill tumor cells and exert chemotherapeutic effects, thus enhancing the therapeutic effect. Furthermore, the synergistic therapy of SDT and chemotherapy could induce pyroptosis of gastric cancer cells and play anti-tumor effects *in vivo* and *in vitro*. Our results may provide a potential method for gastric cancer therapy.

## Data Availability

The original contributions presented in the study are included in the article/Supplementary Material, further inquiries can be directed to the corresponding author.
