# Correction: Biomimetic metal-organic framework nanoparticles for synergistic combining of SDT-chemotherapy induce pyroptosis in gastric cancer

**DOI:** 10.3389/fbioe.2025.1644914

**Published:** 2025-11-14

**Authors:** Zhu Yu, Wenlong Cao, Chuangye Han, Zhen Wang, Yue Qiu, Jiancheng Wang, Mengda Wei, Junfu Wang, Siwen Zhang, Senfeng Liu, Shutian Mo, Junqiang Chen

**Affiliations:** 1 Department of Gastrointestinal Surgery, The First Affiliated Hospital of Guangxi Medical University, Nanning, China; 2 Guangxi Key Laboratory of Enhanced Recovery After Surgery for Gastrointestinal Cancer, Nanning, China; 3 Department of Hepatobiliary Surgery, The First Affiliated Hospital of Guangxi Medical University, Nanning, China

**Keywords:** ZIF-8, sonodynamic therapy, chemotherapy, gastric cancer, pyroptosis

There was a mistake in Figure 5 as published. Figure 5G, the statistical chart of Figure 5C, was inadvertently misplaced during the final formatting process. The corrected Figure 5 appears below.

**FIGURE 5:**
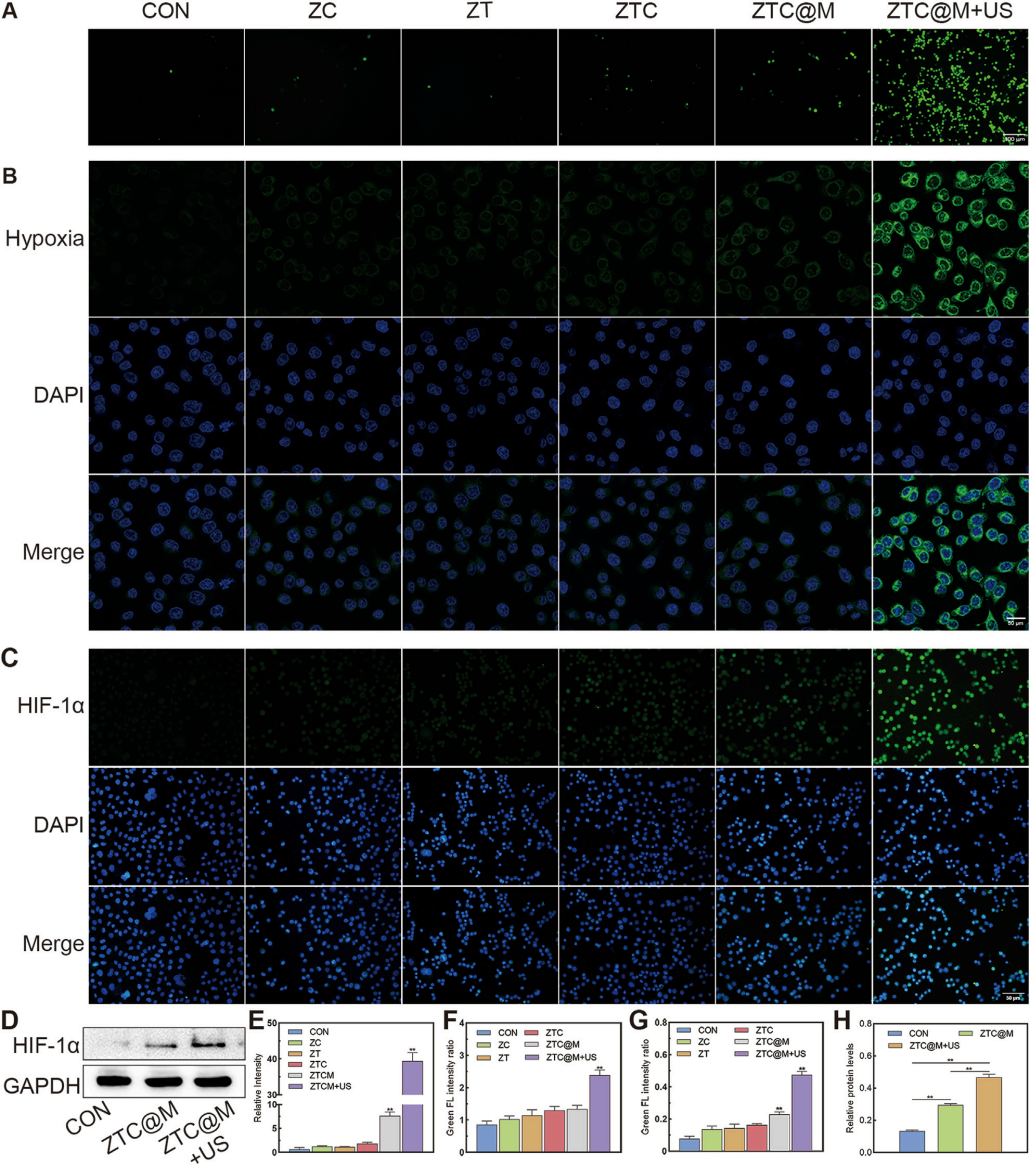
*In vitro* ROS/hypoxia assay and IF. ROS production was detected by fluorescence of DCFH-DA in AGS cells **(A)** (100×). Intracellular hypoxia imaging using Image-iT™ Green Hypoxia Reagent as the syndicator **(B)** (200×). Immunofluorescence images of AGS stained by HIF-1α **(C)** (200×). Gel images of HIF-1α in AGS cells treated with ZTC@M + US **(D)**. Quantification of intracellular fluorescence intensity in A **(E)**, B **(F)**, and C **(G)**. The bar chart indicates the relative density of HIF-1α to GAPDH in D **(H)**. *p < 0.05, **p < 0.0001.

The original article has been updated.

