# Supplementary material for: Biomimetic Metal-Organic Framework Nanoparticles for Synergistic Combining of SDT-Chemotherapy Induce Pyroptosis in Gastric Cancer
Source: Front Bioeng Biotechnol. 2022 Feb 21;10:796820. doi: 10.3389/fbioe.2022.796820 (PMC8899015; doi:10.3389/fbioe.2022.796820)
Supplement: Supplementary file 1 [file Table1.DOCX]

The download link of raw data.

<https://www.jianguoyun.com/p/DZh1_MAQv-z3CRiskJQE>
